# Expression of *microRNA-181a* and *microRNA-196b* in Egyptian Pediatric acute Lymphoblastic Leukemia

**DOI:** 10.31557/APJCP.2020.21.11.3429

**Published:** 2020-11

**Authors:** Roxan Ezzat Shafik, Nahed Abd El Wahab, Marwa M Mokhtar, Maha A El Taweel, Emad Ebeid

**Affiliations:** 1 *Department of Clinical Pathology, NCI, Cairo University, Cairo, Egypt. *; 2 *Department of Pediatric Oncology, NCI, Cairo University, Cairo, Egypt. *

**Keywords:** microRNA, 181a, microRNA, 196b- ALL, pediatric

## Abstract

**Background:**

Differential expression of miRNA provides important insights into pathogenesis of cancer including leukemia. Deregulation of microRNA may contribute to hematopoietic malignancies. In this study, we aimed to evaluate the role of miR-181a and miR-196b in acute lymphoblastic leukemia (ALL) and correlate their expression with clinical and laboratory data.

**Methods:**

The study was performed on bone marrow samples of 70 consecutive newly diagnosed pediatric (ALL) patients, of which 56 were evaluated for both* miR-181a* and *miR-196b* (all 70 for miR-181a) by real-time quantitative reverse transcriptase polymerase chain reaction (RT-qPCR). In addition, bone marrow from seven age and sex matched healthy controls derived from donors of bone marrow transplantation were assessed.

**Results:**

*miR-181a *expression was significantly up-regulated in ALL patients compared with healthy controls (p<0.001). However, *miR-196b* expression was significantly down-regulated in patients compared with healthy controls (p=0.038).

**Conclusion:**

Our results suggest that miR-181a has an oncogenic, while miR-196b has a tumor suppressive role in pediatric ALL patients. A finding which demonstrate the potential role of these microRNAs in pathogenesis of pediatric ALL. Also, estimation of their expression level may provide a tool for confirmation of a diagnosis of childhood ALL and could be a possible predictor of early relapse.

## Introduction

There is general recognition that cancer arises as a result of accumulation of genetic alterations that either activate proto-oncogenes or inactivate tumor suppressor genes (Kent and Mendell, 2006). Recently, a new era that includes a species of non-coding RNAs known as microRNAs (miRNAs) that post-transcriptionally regulate the expression of many protein-coding genes by binding to their targeted mRNA (Jackson and Standart, 2007) Subsequently, the bound mRNA is cleaved or its translation into protein is repressed. They play an important role in many biological processes such as cell proliferation, differentiation and apoptosis (Winter et al., 2009). Their aberrant expression contributes to cellular transformation and tumorigenesis (Calin et al., 2002; Metzler et al., 2004).

*MicroRNA-181a* has been recognized as an important regulator in leukocyte cell differentiation and function(Sun et al., 2014). It was involved in regulating the differentiation of B cells, T cells and natural killer during normal hematopoiesis (Cichocki et al., 2011). It has a key role in T cell maturation (Neilson et al., 2007). MiR-181 family has four members *(miR-181a, miR-181b, miR-181c* and *miR-181d*) (Lim et al., 2005).

MiR-181a was believed to have a dual behavior, acting as a tumor suppressor in glioma (Shi et al., 2008) and in oral squamous cell carcinoma (Shin et al., 2011), while acting as an onco-miRNA in non-small-cell lung cancer (Gao et al., 2010), breast cancer (Taylor et al., 2013), hepatocellular carcinoma (Meng et al., 2012) and gastric and colon cancer (Wei et al., 2015; Zhang et al., 2012).

However, the exact role of miR-181a in leukemia is controversial, some studies suggest that miR-181a act as an onco-miRNA (Luan et al., 2015; Verduci et al., 2018)and others report its role as a tumor suppressor gene (Fragoso et al., 2012; Weng et al., 2015). 

*MiR-196b* is encoded in the HOXA-cluster between the *HOXA9 *and *HOXA10* genes (Schotte et al., 2010). *HOXA* genes is generally known to be involved in survival and proliferation rates of leukemia cells (Orlovsky et al., 2011), *miR-196b* is also overexpressed in bone marrow progenitor cells leading to increase in proliferative capacity and affecting survival (Popovic et al., 2009). However, the role of miR-196b in leukemia is still controversial, as it is involved also in the regulation of oncoproteins such as ERG and c-myc, suggesting a tumor suppressive activity (Bhatia et al., 2010; Coskun et al., 2011).

Based on this information, we aimed to evaluate the expression of* miR-181a* and *miR-196b* in Egyptian childhood acute lymphoblastic leukemia (ALL) and their actual role in pathogenesis of the disease wither having oncogenic or tumor suppressive role and correlate their expression with the clinical and laboratory data.

## Materials and Methods

*Patients and methods*

This study was carried on 70 consecutive newly diagnosed pediatric ALL patients who presented to the pediatric Medical Oncology Department, National Cancer Institute (NCI) over a period of two years. Seven age and sex matched healthy children taken as control group from donors of bone marrow transplantation. 

Diagnosis was established after clinical, morphological, cytochemical, flow cytometric and cytogenetic analysis. All the cases met the ALL diagnosis standards. A Written informed consent (obtained from guardians of the children aged 1 to 18 years) was approved by the Institutional Review board (IRB) ethical committee of the NCI which follows the rules of Helsinki IRB. 

Inclusion criteria: (1) De novo acute lymphoblastic leukemia patients. (2) Either sex was eligible. (3) Age: 1-18 years. (4) Egyptians patients. Exclusion criteria: (1) Treated ALL patients. (2) Adult age group. (3) Non-Egyptians. 

*Sample collection, RNA preparation and cDNA synthesis*

Bone marrow samples (1 ml) were collected on EDTA from pediatric ALL patients and controls taken from bone marrow donors for bone marrow transplantation. Bone marrow was treated with erythrocytes lysis solution. Leukocytes were collected and stored in QIAzol lysis reagent at -80°C till use for RNA extraction. 

Total RNA was extracted from mononuclear cells using miRNeasy Mini kit (QIAGEN), following the manufacturer’s instructions. The amount of RNA was measured by nanodrop spectrophotometer at 260 and 280 wave length; (a ratio of 1.8-2.1) denoted good quality of RNA. Subsequently,1.0 μg of total RNA was reverse transcribed into cDNA in 20 μL reaction using random hexamer using miScript II RT kit (QIAGEN) according to manufacturer’s instructions and stored at -20°C till use. 

*Quantitative Real-time PCR (qPCR) for miRNAs*

The expression of *miRNA* was determined using reverse transcription quantitative real-time polymerase chain reaction (RT-qPCR). For real-time RT-qPCR, the 25 μl reaction contained 12.5 μl 2X Quantitect SYBR GREEN PCR Master Mix (QIAGEN), 5 pmol (2.5 μl) 10X miScript universal primer for either *miR- 181a* or *196b* and 2.5 μl of the diluted cDNA (diluted with 25 μl of Nuclease-free water). Reactions were run with the following thermal cycles parameters: 95°C for 15 minutes, followed by 40 PCR cycles at 94°C (15 seconds, denaturation), 55°C (30 seconds, annealing) and 70°C (30 seconds, extension). Relative expression of* miR-181a *and *196b* was analyzed by the comparative Ct method (2^−ΔΔCt^), using SNORD 68 RNA as the endogenous control. Data were expressed as the fold change in gene expression in the patients normalized to the expression levels of the endogenous control and relative to the healthy controls. 

*Statistical methods*

Statistical analysis was done using IBM^©^ SPSS^© ^version 22. Numerical data were expressed as means and standard deviation or medians and ranges as appropriate. Qualitative data were expressed as frequency and percentage. Chi-square test or Fisher ʼs exact test was used to examine the relation between qualitative variables. For the non-normally distributed data, comparison between two groups was done using Mann-Whitney test (non-parametric t-test). Spearman-rho method was used to test correlation between numerical variables. Survival analysis was done using Kaplan-Meier method and comparison between two survival curves was done using log-rank test. Relation between the expressions of two genes was done using Mc-Nemar test. All tests were two tailed. A p-value < 0.05 was considered significant.

## Results

The study was done on 70 consecutive newly diagnosed Egyptian pediatric acute lymphoblastic leukemia (ALL) patients, out of which, 56 were evaluated for both *miR-181a* and *miR-196b* and all 70 patients were evaluated for *miR-181a* together with seven age and sex matched healthy controls. Expression levels of* miR-181a*, *miR-196b* and SNORD 68 RNA were determined by real-time quantitative reverse transcriptase polymerase chain reaction (RT-qPCR). 

Mean value of expression level of *miR-181a* in control group was 0.015 and this value was taken as a cut off, where patients having values above this cut off were considered as high expressers for *miR-181a* and patients having values below this cut off were considered as low expressers for *miR-181a*. Similarly, the mean value of expression level of *miR-196b* in the control group was 0.001 and this value was taken as cut off for classifying patients as high or low* miR-196b* expressers. (Demographic and laboratory characteristics of patients are summarized in Table 1). 

The expression of* miR-181a *was statistically significantly elevated in ALL patients compared with control group (p< 0.001). However, *miR-196b* expression was significantly down-regulated in ALL patients compared to control group (p=0.038) (Table 2). 

Sixty eight patients (97.1%) out of seventy show high expression for* miR-181a* and only two patients (2.9%) show low expression. However, forty nine patients (87.5%) are *miR-196b* low expressers and seven (12.5%) are high expressers.

No statistically significant differences were encountered between high and low *miR-196b* expressers as regards age, sex, organomegaly or lymphadenopathy. Neither high and low *miR-196b* expressers show significant difference regarding Hb level, TLC, platelet count, peripheral blood, bone marrow blasts, cytogentics or immunophenotyping (Table 3). No statistically significant differences were detected between high and low expressers of *miR-196b *as regards response to induction therapy (p=0.3) (Table 4). 

A positive correlation was observed between *miR-181a* and *miR-196b* expression by Spearman’ rho test with a correlation coefficient=0.344 and p value =0.009.

The median follow up duration was 15.2 months (range 0.03-35.6 months). Median overall survival was not reached. 87.8% of the patients were still alive at 18 months. In our study, eight patients died, out of which 4 show low expression and 1 show high expression for *miR-196b* and three patients couldn’t be evaluated. As regard miR-181a all died patients were high expressers.

## Discussion

MicroRNAs are proved to be involved in regulation of normal hematopoiesis and their dysregulation has been related to various types of cancer, including hematological malignancies. We aimed to analyze the expression levels of *miR-181a* and *miR-196b* in bone marrow samples pediatric ALL patients and their role in pathogenesis of the disease.

In this study, we observed that *miR-181a* has a role of onco-miRNA in ALL. By using qRT-PCR, bone marrow samples of children with ALL showed highly significant increase in expressions levels of *miR-181a *compared to bone marrow samples obtained from healthy controls (P<0.001). This oncogenic theme was consistent with a study (Duyu et al., 2014) that reported increase in expression level of miR-181a in pediatric ALL upon diagnosis that significantly decreased following a six month treatment period.

Similar to our results, another study (Verduci et al., 2015) demonstrated that miR-181a can act as an onco-miRNA by repressing the tumor suppressor EGR1. Also, upregulation of miR-181a might contribute in the development of drug resistance in ALL cell lines (Liao et al., 2015).

Moreover, another study (Yan et al., 2015), found that patients with T cell leukemia/lymphoma showed increase in level of *miR-181a* and this overexpression was associated with increased AKT phosphorylation and can contribute to chemoresistance in T cell leukemia/lymphoma assuming that *miR-181a* could be a promising therapeutic target in treating T-cell malignancies resistant to chemotherapy.

On the contrary, there was a study (Nabhan et al., 2017) that show that the *miR-181a* expression was significantly decreased in serum samples of ALL patients compared to control group. They obtained evidence that *miR-181a* could act as a tumor suppressor through overexpression of its target pair smad7 which act as a negative regulator for the transforming growth factor-B (TGF-B).

Moreover, two studies (Schotte et al., 2010; Yang et al., 2015( reported under-expression of *miR-181a-1* in peripheral blood samples of pediatric ALL carrying t(12;21) translocation. The study demonstrated that miR-181a could target ETV6/RUNX1 oncoprotein and cause reduction in its level. In our study, only three of our patients had t(12;21).

So, the same micro RNA can function as tumor suppressor or oncogene, depending on the cellular context and on the consequent expression of its targets (Verduci et al., 2015). It was reported that the usage of BM samples for miRNA analysis in hematological malignancies reflect the leukemic process more efficiently, when compared to peripheral blood (Duyu et al., 2014).

As regards *miR-196b*, in our study, it was found to be significantly under-expressed in pediatric ALL bone marrow samples compared to bone marrow of healthy controls (P =0.038). Similar to our results, there is a study (de Oliveira et al., 2012) that reported low expression of miR-196b in pediatric ALL bone marrow samples compared to normal pediatric bone marrow samples and down-regulation *miR-196b* was demonstrated in B-ALL without translocations compared to CD34 cells. There are reports of involvement of miR-196b in the regulation of oncoproteins such as ERG and c-myc, suggesting a tumor suppressive activity. 

Moreover, it was observed that miR-196b was not only found to be significantly down-regulated in B-cell ALL patients as compared to that found in the corresponding controls, but also had the inherent capacity to down-regulate the highly expressed c-myc gene. Hence miR-196b can be assigned with the tumor suppressor function and can be of therapeutic importance toward the treatment of B-cell ALL (Bhatia et al., 2010).

However, in patients with chromosomal translocations involving the Mixed Lineage Leukemia (MLL) gene. Leukemogenic MLL fusion protein cause overexpression of miR-196b leading to increased proliferative capacity and survival, as well as a partial block in differentiation. So miR-196b is overexpressed in primary leukemia samples from MLL patients, but not from other types of leukemia. (Popovic et al., 2009). In our study, none of patients had translocations involving MLL gene.

In conclusion, the discovery of microRNAs and their association with disease have provided valuable information on potential diagnostic and/or prognostic biomarkers, as well as monitoring the disease progression. In our study, *miR-181a* was found to be significantly elevated in pediatric ALL patients which suggests its role as an onco-miRNA. While, miR-196b was significantly depressed in ALL patients and can be assigned as a tumor suppressor microRNA. These findings suggest a potential role of these microRNAs in pediatric ALL, which can predict clinical outcome, risk of relapse and also can be a novel target for cancer therapy. Further studies are needed to corroborate and extend our results.

**Table 1:** Clinical and Laboratory Characteristics of 70 Pediatric Acute Lymphoblastic Leukemia

Parameter	Findings
Age	7.3 ± 5.1*
< 2 years	11 (15.7)**
≥ 2 years	59 (84.3)**
Sex	
Male	37 (52.9)**
Female	33 (47.1)**
Hepatomegaly	42 (60)**
Splenomegaly	41 (58.6)**
Lymphadenopathy	37 (52.9)**
Total leukocytic count x 109/L	86.49 ± 154.41*
< 50 X 10^9^/L	49 (70)**
≥ 50 X 10^9^/L	21 (30)**
Hemoglobin gm/dl	8.01 ± 2.1*
<7 gm/dl	23 (32.9)**
≥7 gm/dl	47 (67.1)**
Platelet x 10^9^/L	81.08 ± 118.14*
<100	57 (81.4)**
≥100	13 (18.6)**
Peripheral blood blasts	54.3 ± 33.4*
<10	13(18.6)**
≥ 10	57 (81.4)**
Bone marrow blasts	87.3 ± 12.40*
< 90	23 (32.9)**
≥ 90	47 (67.1)**
Bone marrow cellularity	
Normocellular	14 (20)**
Hypercellular	53 (75.7)**
Hypocellular	3 (1.4)**
Cytogenetics and molecular	
Normal karyotype	56 (80)**
Hyperdiploidy	5 (7.1)**
t (1;19)	1 (1.4)**
t (9;22)	5 (7.1)**
t (12;21)	3 (4.3)**
Immunophentyping	
B-phenotype	57(81.4)**
Pre-B	43 (61.4)**
Common-ALL	14 (20)**
T-phenotype	
Early-T	8 (11.4)**
Intermediate-T	3 (4.3)**
Late-T	2 (2.9)**
CD 34	
Positive	39 (55.7)**
Negative	31 (44.3)**

**Table 2:** Expression of miRNA-181a and *miRNA-196b *in Pediatric Acute Lymphoblastic Leukemia Patients versus Control Group

Gene expression	ALL group	Control group	P-value
MiRNA-181a	(n=70)	(n=7)	<0.001
Over expression	68 (97.1)**	3 (42.9)**	
Under expression	2 (2.9)**	4 (57.1)**	
MiRNA-196b	(n=56)	(n=7)	0.038
Over expression	7 (12.5)**	3 (42.86)**	
Under expression	49 (87.5)**	4 (57.14)**	

**Number (%)

**Table 3:** Characteristics of Pediatric ALL Patients According to *miRNA-196b* and *miRNA-181a* Expression Levels

	miRNA-196b		P-value	miRNA-181a	Over expression (n=68)
	Under expression (n=49)	Over expression (n=7)		Under expression (n=2)	
Age			0.336		
< 2 years	7 (14.2)**	2 (28.5)**		0 (0.0)**	11 (16.2)**
≥ 2 years	42 (85.7)**	5 (71.4)**		2 (100)**	57 (83.8)**
Sex			1		
Male	27 (55.1)**	4 (57.1)**		0 (0.0)**	37 (54.4)**
Female	22 (44.8)**	3 (42.8)**		2 (100)**	31 (45.5)**
Hepatomegaly	26 (53.1)**	6 (85.7)**	0.219	2 (100)**	40 (58.9)**
Splenomegaly	27 (55.1)**	3 (42.8)**	0.693	2 (100)**	39 (57.3)**
Lymphadenopathy	27 (55.1)**	4 (57.1)**	1	0 (0.0)**	37 (54.4)**
Total leukocytic count x 10^9^/L			0.421		
< 50 X 10^9^/L	33 (67.3)**	6 (85.7)**		0 (0.0)**	49 (72.1)**
≥ 50 X 10^9^/L	16 (32.6)**	1 (14.2)**		2 (100)**	19 (27.9)**
Hemoglobin gm/dl			0.909		
<7 gm/dl	13 (26.5)**	2 (28.5)**		1 (50)**	22 (32.2)**
≥7 gm/dl	36 (73.4)**	5 (71.4)**		1 (50)**	46 (67.6)**
Platelet x 10^9^/L			0.703		
<100	39 (79.5)**	6 (85.7)**		2 (100)**	55 (80.8)**
≥100	10 (20.4)**	1 (14.2)**		0 (0.0)**	13 (19.1)**
Peripheral blood blasts	54.7±34.1*	43.6±33.0*	0.367	79.0±22.6*	53.5±33.5*
Bone marrow blasts	87.9±10.2*	89.7±11.1*	0.23	91.0±1.4*	87.2±12.6*
Bone marrow cellularity			0.816		
Normocellular versus hypocellular	12 (24.4)**	2 (28.5)**		0 (0.0)**	17 (25)**
Hypercellular	37 (75.5)**	5 (71.4)**		2 (100)**	51 (75)**
Immunophenotyping			0.7		
B-ALL	39 (79.5)**	6 (85.7)**		0 (0.0)**	57 (83.8)**
T-ALL	10 (20.4)**	1 (14.2)**		2 (100)**	11 (16.1)**
CD34			0.431		
Positive	25 (51)**	5 (71.4)**		1 (50)**	30 (44.1)**
Negative	24 (48.9)**	2 (28.5)**		1 (50)**	38 (55.8)**
Cytogenetics					
Hyperdiploidy	4 (8.1)**	0	no	0	5 (7.3)**
t (1;19)	1 (2.04)**	0	no	0	5 (7.3)**
t (9;22)	5 (10.2)**	0	1	0	3 (4.4)**
t (12;21)	0	1 (14.2)	No	0	3 (4.4)**

*, Mean ± SD, **, Number (%); No P-value regarding miR-181a expression because only 2 cases were under-expressed.

**Table 4:** Response to Induction Therapy According to *miR-181a *and *miR-196b* Expression Levels in Pediatric ALL Patients

	miR-196b		P-value	miR-181a	
	Under expression (n=46)	Over expression (n=7)		Under expression (n=2)	Over expression (n=64)
Hematological response			0.185		
Complete remission	40 (86.9)**	7 (100)**		1 (50)**	56 (87.5)**
Not in Complete remission	6 (13.0)**	0 (0.0)**		1 (50)**	8 (12.5)**
Minimal Residual Disease (MRD)			0.453		
Complete remission	24 (52.3)**	4 (57.1)**		0 (0.0)**	34 (53.1)**
Not in Complete remission	22 (47.8)**	3 (42.8)**		2 (100)**	30 (46.8)**

No P-value for comparisons of *miR-181a *expressions because only 2 cases were under-expressed.
